# Association Between Peripheral Nerve Blocks and Reduced Opioid Rescue Use in the PACU After Knee Surgery: A Retrospective Study From a Mexican Tertiary Center

**DOI:** 10.7759/cureus.89055

**Published:** 2025-07-30

**Authors:** Roberto de Jesus Jiménez Contreras, Lourdes Trinidad Castillo Garcia, Mario Alberto Quintero García, María Fernanda Figueroa Caballero

**Affiliations:** 1 Anesthesiology, American British Cowdray (ABC) Medical Center, Mexico City, MEX

**Keywords:** knee surgery, opioid-sparing anesthesia, peripheral nerve block, post-anesthesia care unit, postoperative rescue analgesia

## Abstract

Introduction: Peripheral nerve blocks (PNBs) are increasingly used for multimodal analgesia in knee surgery, yet their impact on opioid use in the recovery room remains understudied. This study aimed to assess the effectiveness of PNBs in being associated with reduced need for opioid rescue in the post-anesthesia care unit (PACU).

Methods: This retrospective cohort study included 123 patients who underwent knee surgery under general anesthesia with or without the addition of a peripheral nerve block. The primary outcome was the requirement of intravenous opioid rescue during PACU stay. Data were analyzed using chi-square and Fisher's exact tests.

Results: Of the total patients, 61 received general anesthesia alone and 62 received general anesthesia plus a PNB. Opioid rescue in the PACU was required in 27.87% of patients without a PNB versus 12.90% with a PNB (p = 0.043, chi-square = 4.09). This corresponds to a relative risk reduction of 53.7%, an absolute risk reduction of 14.97%, and a number needed to treat (NNT) of 7.

Conclusion: The use of peripheral nerve blocks in knee surgery is associated with a significant reduction in the need for opioid rescue in the PACU. These findings support the incorporation of PNBs into standard multimodal analgesia protocols.

## Introduction

Knee surgery, whether arthroscopic or reconstructive, is frequently associated with moderate to severe postoperative pain. Inadequately controlled pain can delay mobilization, prolong hospitalization, and hinder functional recovery. Traditionally, systemic opioids have been the mainstay of postoperative analgesia; however, their side effects, such as nausea, vomiting, respiratory depression, and the risk of dependence, have led to the adoption of multimodal pain management strategies [1,2].

Peripheral nerve blocks (PNBs) have become a key element in multimodal analgesia. Techniques including femoral, adductor canal, and sciatic nerve blocks have demonstrated significant benefits in reducing opioid consumption and improving patient satisfaction after knee surgery [3-5]. Moreover, PNBs are integral to enhanced recovery after surgery (ERAS) protocols widely implemented in orthopedic settings [6,7].

Despite strong evidence supporting the use of PNBs in North American and European contexts, there is limited real-world data from Latin American institutions regarding their effectiveness in the immediate postoperative setting [8]. This study aims to evaluate whether the use of PNBs in patients undergoing knee surgery under general anesthesia is associated with a reduced need for intravenous opioid rescue in the post-anesthesia care unit (PACU) at a tertiary care center in Mexico.

## Materials and methods

This was a retrospective, observational, comparative study conducted at a tertiary-level private hospital in Mexico City. The protocol was approved by the Institutional Ethics and Research Committees of American British Cowdray (ABC) Medical Center under reference code TABC-22-13. Patient confidentiality was preserved throughout the study, and no personally identifiable data were included.

A total of 123 adult patients who underwent elective knee surgery between January and December 2019 were included. All patients received general anesthesia as the primary anesthetic technique. They were divided into two groups according to whether a peripheral nerve block (PNB) was performed in addition to general anesthesia. The "no PNB group" (n = 61) included patients who received general anesthesia only, while the "PNB group" (n = 62) received general anesthesia plus a PNB, which could include femoral, adductor canal, or sciatic nerve blocks, based on the anesthesiologist's discretion and surgical indication.

Patients with incomplete anesthetic records, those who received regional anesthesia without general anesthesia, and pediatric patients were excluded.

The primary outcome was the need for intravenous opioid rescue (such as fentanyl or morphine) administered in the post-anesthesia care unit (PACU). Secondary variables included age, sex, American Society of Anesthesiologists (ASA) physical status, type of surgical procedure (e.g., arthroscopy versus open surgery), and anesthetic duration. All data were obtained from the institutional electronic medical records system and reviewed manually to ensure accuracy.

Data were recorded in a standardized data sheet and analyzed using IBM SPSS Statistics version 25 (IBM Corp., Armonk, NY). Categorical variables were expressed as absolute values and percentages, and compared using the chi-square test or Fisher's exact test, as appropriate. Continuous variables were presented as means ± standard deviations (SD) and compared using Student's t-test. A p-value < 0.05 was considered statistically significant.

## Results

A total of 123 patients met the inclusion criteria and were analyzed. Of these, 61 patients received general anesthesia only (no PNB group), and 62 received general anesthesia in combination with a peripheral nerve block (PNB group). Baseline demographic and clinical characteristics were statistically similar between groups, as shown in Table 1.

**Table 1:** Demographic and Clinical Characteristics of Patients Undergoing Knee Surgery Demographic and baseline clinical data were comparable between groups. PNB: peripheral nerve block, SD: standard deviation, ASA: American Society of Anesthesiologists

Variable	No PNB (n = 61)	PNB (n = 62)	p-value
Age (years), mean ± SD	53.4 ± 12.1	52.7 ± 11.9	0.75
Male sex, number (%)	35 (57.4%)	33 (53.2%)	0.65
ASA I-II, number (%)	52 (85.2%)	50 (80.6%)	0.48
Arthroscopy procedure, number (%)	28 (45.9%)	30 (48.4%)	0.77
Anesthesia time (minutes), mean ± SD	98 ± 16	100 ± 14	0.48

The mean age of patients in the no PNB group was 53.4 ± 12.1 years, compared to 52.7 ± 11.9 years in the PNB group (p = 0.75). The proportion of male patients was also similar (57.4% versus 53.2%, p = 0.65). There were no statistically significant differences in ASA physical status classification, the proportion of patients undergoing arthroscopy, or anesthetic duration between the two groups (all p > 0.05). These findings confirm appropriate group comparability prior to outcome assessment, which is critical in retrospective comparative analyses [1,2].

The primary outcome (requirement for intravenous opioid rescue in the post-anesthesia care unit (PACU)) differed significantly between the groups. In the no PNB group, 17 of 61 (27.87%) patients required opioid rescue, whereas in the PNB group, only eight of 62 (12.90%) patients did (p = 0.043, Fisher's exact test p = 0.049), indicating a statistically significant reduction associated with the use of PNBs. This corresponds to a relative risk reduction (RRR) of 53.7% and an absolute risk reduction (ARR) of 14.97%, resulting in a number needed to treat (NNT) of approximately 7 to prevent one additional opioid rescue event in the PACU.

This reduction in immediate opioid requirement is consistent with prior evidence supporting the effectiveness of femoral and adductor canal blocks in total knee arthroplasty and arthroscopy [3,4]. Several meta-analyses and clinical trials have shown that regional blocks significantly reduce postoperative pain intensity and opioid consumption, contributing to improved recovery and fewer opioid-related side effects [5-7].

Furthermore, our findings reinforce the integration of regional techniques into enhanced recovery protocols, where reduced PACU opioid administration has been correlated with earlier mobilization, improved patient satisfaction, and shorter PACU stays [9,10].

No adverse events related to peripheral nerve block administration were recorded in our study cohort, supporting the overall safety of these interventions when performed by trained anesthesiologists using ultrasound guidance [11].

## Discussion

The findings of this study demonstrate that the use of peripheral nerve blocks (PNBs), in addition to general anesthesia, is associated with a significant reduction in the need for intravenous opioid rescue during the immediate postoperative period in patients undergoing knee surgery. Specifically, only 12.9% of patients in the PNB group required rescue opioids in the PACU compared to 27.9% in the group receiving general anesthesia alone, supporting the analgesic effectiveness of regional blocks in orthopedic surgery.

These results are consistent with prior literature indicating that peripheral nerve blocks, including femoral and adductor canal blocks, provide effective analgesia for knee procedures and significantly reduce opioid consumption [3-5]. Ilfeld et al. conducted a comprehensive review of continuous PNBs and found a consistent association with reduced pain scores, opioid requirements, and related adverse effects across various orthopedic surgeries [3].

Moreover, our study aligns with the findings of Chan et al., who reported in a Cochrane meta-analysis that femoral nerve blocks significantly decreased pain and opioid use after total knee arthroplasty, with fewer systemic complications [4]. Similarly, Kehlet and Dahl advocated for multimodal analgesia strategies, including regional techniques, as key to enhancing postoperative recovery while minimizing opioid-related side effects [1].

The clinical implications of reduced opioid use are important. Opioid-related adverse effects such as nausea, vomiting, respiratory depression, and prolonged PACU stay are well documented and may hinder recovery [1,7]. By minimizing opioid exposure through effective regional techniques, clinicians can improve patient satisfaction and recovery timelines while contributing to broader institutional goals of opioid stewardship [9,10].

Enhanced recovery after surgery (ERAS) protocols strongly recommend incorporating regional anesthesia whenever possible. Schwenk and Mariano noted that regional blocks form a recommended component in ERAS protocols, helping reduce opioid dependence and accelerating functional recovery [7]. The present study supports these recommendations within a Latin American tertiary care setting, where regional anesthesia adoption is increasing but still variable in clinical practice.

Importantly, no adverse effects related to PNB were observed in our cohort, corroborating findings from the studies by Liu et al. [5] and Rawal [6], who demonstrated that when performed by trained practitioners using ultrasound guidance, single-shot nerve blocks are both safe and effective [12].

Nonetheless, several limitations should be acknowledged. This study was retrospective in nature and thus subject to potential confounding factors. Pain scores were not consistently recorded in the medical records, limiting direct assessment of analgesic efficacy beyond opioid consumption. The variability in nerve block technique (e.g., type of block, volume, and timing) was not fully standardized, which could influence outcomes. Additionally, the study did not evaluate long-term functional outcomes or time to ambulation, which would provide a more comprehensive assessment of recovery quality.

Furthermore, detailed information on actual opioid dosages (e.g., morphine equivalents) and time to first rescue analgesia was not available in the electronic records. These data limitations, inherent to the retrospective design, precluded a more granular analysis of analgesic effectiveness. Future prospective studies should address this gap by standardizing documentation of drug doses, block type, and pain scores to enable more precise comparisons.

Despite these limitations, the study provides valuable real-world evidence from a Latin American population supporting the use of peripheral nerve blocks in enhancing postoperative recovery after knee surgery. These findings reinforce the importance of incorporating regional techniques into everyday anesthetic practice, especially in orthopedic procedures where pain control is paramount.

## Conclusions

The use of peripheral nerve blocks in patients undergoing knee surgery under general anesthesia was associated with a significant reduction in the need for intravenous opioid rescue during the immediate postoperative period in the PACU. These findings support the incorporation of peripheral nerve blocks as a recommended component of multimodal analgesia protocols for knee surgery, particularly in settings aiming to reduce opioid exposure and improve recovery quality. While further prospective studies are needed to confirm these benefits and assess long-term outcomes, the present study adds to the growing body of evidence favoring regional anesthesia techniques in orthopedic surgical care.
